# Supplementary material for: Attitudes towards Italian Mafias Scale (AIMS): development and validation
Source: PeerJ. 2023 Oct 24;11:e16120. doi: 10.7717/peerj.16120 (PMC10607589; doi:10.7717/peerj.16120)
Supplement: Supplemental Information 4 — JASP (https://jasp-stats.org/) script to perform parametric, non-parametric analyses both for frequentist and bayesian approach for testing the difference between DRMR participants and those from the rest of Italy in Study 2 [file peerj-11-16120-s004.jasp › index.html]

JASP 


# Results

## Independent Samples T-Test

| Independent Samples T-Test | | | | | | | | | | | | | | | |
| --- | --- | --- | --- | --- | --- | --- | --- | --- | --- | --- | --- | --- | --- | --- | --- |
|  | | | | | | | | | | | | 95% CI for Effect Size | | | |
|  | | Test | | Statistic | | df | | p | | Effect Size | | Lower | | Upper | |
| fscoresAIMS.collapsed |  | Student |  | -3.113 |  | 391 |  | 0.002 |  | -0.314 |  | -0.513 |  | -0.115 |  |
|  |  | Mann-Whitney |  | 15407.500 |  |  |  | < .001 |  | -0.202 |  | -0.309 |  | -0.090 |  |
| AIMS\_mean\_collapsed |  | Student |  | -2.145 |  | 391 |  | 0.033 |  | -0.216 |  | -0.415 |  | -0.018 |  |
|  |  | Mann-Whitney |  | 16554.500 |  |  |  | 0.015 |  | -0.142 |  | -0.252 |  | -0.029 |  |
| fscoresAIMS.Notcollapsed |  | Student |  | -2.900 |  | 391 |  | 0.004 |  | -0.293 |  | -0.491 |  | -0.094 |  |
|  |  | Mann-Whitney |  | 15633.500 |  |  |  | 0.001 |  | -0.190 |  | -0.298 |  | -0.078 |  |
| AIMS\_mean\_NOTcollapsed |  | Student |  | -1.987 |  | 391 |  | 0.048 |  | -0.200 |  | -0.399 |  | -0.002 |  |
|  |  | Mann-Whitney |  | 16693.500 |  |  |  | 0.020 |  | -0.135 |  | -0.245 |  | -0.022 |  |
|  | | | | | | | | | | | | | | | |
|  |  |  |  |  |  |  |  |  |  |  |  |  |  |  |  |
| --- | --- | --- | --- | --- | --- | --- | --- | --- | --- | --- | --- | --- | --- | --- | --- |
| *Note.*  For the Student t-test, effect size is given by Cohen's d. For the Mann-Whitney test, effect size is given by the rank biserial correlation. | | | | | | | | | | | | | | | |

### Assumption Checks

| Test of Normality (Shapiro-Wilk) | | | | | | | |
| --- | --- | --- | --- | --- | --- | --- | --- |
|  | |  | | W | | p | |
| fscoresAIMS.collapsed |  | 1 |  | 0.994 |  | 0.582 |  |
|  |  | 2 |  | 0.976 |  | 0.002 |  |
| AIMS\_mean\_collapsed |  | 1 |  | 0.976 |  | 0.002 |  |
|  |  | 2 |  | 0.992 |  | 0.391 |  |
| fscoresAIMS.Notcollapsed |  | 1 |  | 0.994 |  | 0.585 |  |
|  |  | 2 |  | 0.975 |  | 0.001 |  |
| AIMS\_mean\_NOTcollapsed |  | 1 |  | 0.973 |  | < .001 |  |
|  |  | 2 |  | 0.989 |  | 0.118 |  |
|  | | | | | | | |
|  |  |  |  |  |  |  |  |
| --- | --- | --- | --- | --- | --- | --- | --- |
| *Note.*  Significant results suggest a deviation from normality. | | | | | | | |

| Test of Equality of Variances (Levene's) | | | | | | | | | |
| --- | --- | --- | --- | --- | --- | --- | --- | --- | --- |
|  | | F | | df1 | | df2 | | p | |
| fscoresAIMS.collapsed |  | 1.656 |  | 1 |  | 391 |  | 0.199 |  |
| AIMS\_mean\_collapsed |  | 0.464 |  | 1 |  | 391 |  | 0.496 |  |
| fscoresAIMS.Notcollapsed |  | 2.293 |  | 1 |  | 391 |  | 0.131 |  |
| AIMS\_mean\_NOTcollapsed |  | 0.511 |  | 1 |  | 391 |  | 0.475 |  |
|  | | | | | | | | | |

### Descriptives

| Group Descriptives | | | | | | | | | | | | | |
| --- | --- | --- | --- | --- | --- | --- | --- | --- | --- | --- | --- | --- | --- |
|  | | Group | | N | | Mean | | SD | | SE | | Coefficient of variation | |
| fscoresAIMS.collapsed |  | 1 |  | 198 |  | -0.135 |  | 0.920 |  | 0.065 |  | -6.828 |  |
|  |  | 2 |  | 195 |  | 0.141 |  | 0.831 |  | 0.060 |  | 5.907 |  |
| AIMS\_mean\_collapsed |  | 1 |  | 198 |  | 2.430 |  | 0.785 |  | 0.056 |  | 0.323 |  |
|  |  | 2 |  | 195 |  | 2.595 |  | 0.744 |  | 0.053 |  | 0.287 |  |
| fscoresAIMS.Notcollapsed |  | 1 |  | 198 |  | -0.123 |  | 0.928 |  | 0.066 |  | -7.521 |  |
|  |  | 2 |  | 195 |  | 0.134 |  | 0.826 |  | 0.059 |  | 6.178 |  |
| AIMS\_mean\_NOTcollapsed |  | 1 |  | 198 |  | 2.456 |  | 0.810 |  | 0.058 |  | 0.330 |  |
|  |  | 2 |  | 195 |  | 2.614 |  | 0.766 |  | 0.055 |  | 0.293 |  |
|  | | | | | | | | | | | | | |

## Bayesian Independent Samples T-Test- non parametric

| Bayesian Mann-Whitney U Test | | | | | | | |
| --- | --- | --- | --- | --- | --- | --- | --- |
|  | | BF₁₀ | | W | | Rhat | |
| AIMS\_mean\_collapsed |  | 1.121 |  | 16554.500 |  | 1.005 |  |
| fscoresAIMS.collapsed |  | 8.041 |  | 15407.500 |  | 1.002 |  |
| fscoresAIMS.Notcollapsed |  | 4.226 |  | 15633.500 |  | 1.003 |  |
| AIMS\_mean\_NOTcollapsed |  | 0.836 |  | 16693.500 |  | 1.000 |  |
|  | | | | | | | |
|  |  |  |  |  |  |  |  |
| --- | --- | --- | --- | --- | --- | --- | --- |
| *Note.*  Result based on data augmentation algorithm with 5 chains of 2000 iterations. | | | | | | | |

| Descriptives | | | | | | | | | | | | | | | | | |
| --- | --- | --- | --- | --- | --- | --- | --- | --- | --- | --- | --- | --- | --- | --- | --- | --- | --- |
|  | | | | | | | | | | | | | | 95% Credible Interval | | | |
|  | | Group | | N | | Mean | | SD | | SE | | Coefficient of variation | | Lower | | Upper | |
| AIMS\_mean\_collapsed |  | 1 |  | 198 |  | 2.430 |  | 0.785 |  | 0.056 |  | 0.323 |  | 2.320 |  | 2.540 |  |
|  |  | 2 |  | 195 |  | 2.595 |  | 0.744 |  | 0.053 |  | 0.287 |  | 2.490 |  | 2.701 |  |
| fscoresAIMS.collapsed |  | 1 |  | 198 |  | -0.135 |  | 0.920 |  | 0.065 |  | -6.828 |  | -0.264 |  | -0.006 |  |
|  |  | 2 |  | 195 |  | 0.141 |  | 0.831 |  | 0.060 |  | 5.907 |  | 0.023 |  | 0.258 |  |
| fscoresAIMS.Notcollapsed |  | 1 |  | 198 |  | -0.123 |  | 0.928 |  | 0.066 |  | -7.521 |  | -0.253 |  | 0.007 |  |
|  |  | 2 |  | 195 |  | 0.134 |  | 0.826 |  | 0.059 |  | 6.178 |  | 0.017 |  | 0.251 |  |
| AIMS\_mean\_NOTcollapsed |  | 1 |  | 198 |  | 2.456 |  | 0.810 |  | 0.058 |  | 0.330 |  | 2.342 |  | 2.569 |  |
|  |  | 2 |  | 195 |  | 2.614 |  | 0.766 |  | 0.055 |  | 0.293 |  | 2.506 |  | 2.722 |  |
|  | | | | | | | | | | | | | | | | | |

### Descriptives Plots

#### AIMS\_mean\_collapsed

#### fscoresAIMS.collapsed

#### fscoresAIMS.Notcollapsed

#### AIMS\_mean\_NOTcollapsed

## Bayesian Independent Samples T-Test - parametric

| Bayesian Independent Samples T-Test | | | | | |
| --- | --- | --- | --- | --- | --- |
|  | | BF₁₀ | | error % | |
| AIMS\_mean\_collapsed |  | 1.019 |  | 0.019 |  |
| fscoresAIMS.collapsed |  | 11.462 |  | 0.002 |  |
| AIMS\_mean\_NOTcollapsed |  | 0.745 |  | 0.025 |  |
| fscoresAIMS.Notcollapsed |  | 6.244 |  | 0.004 |  |
|  | | | | | |

### Inferential Plots

#### AIMS\_mean\_collapsed

##### Bayes Factor Robustness Check

#### fscoresAIMS.collapsed

##### Bayes Factor Robustness Check

#### AIMS\_mean\_NOTcollapsed

##### Bayes Factor Robustness Check

#### fscoresAIMS.Notcollapsed

##### Bayes Factor Robustness Check

| Descriptives | | | | | | | | | | | | | | | | | |
| --- | --- | --- | --- | --- | --- | --- | --- | --- | --- | --- | --- | --- | --- | --- | --- | --- | --- |
|  | | | | | | | | | | | | | | 95% Credible Interval | | | |
|  | | Group | | N | | Mean | | SD | | SE | | Coefficient of variation | | Lower | | Upper | |
| AIMS\_mean\_collapsed |  | 1 |  | 198 |  | 2.430 |  | 0.785 |  | 0.056 |  | 0.323 |  | 2.320 |  | 2.540 |  |
|  |  | 2 |  | 195 |  | 2.595 |  | 0.744 |  | 0.053 |  | 0.287 |  | 2.490 |  | 2.701 |  |
| fscoresAIMS.collapsed |  | 1 |  | 198 |  | -0.135 |  | 0.920 |  | 0.065 |  | -6.828 |  | -0.264 |  | -0.006 |  |
|  |  | 2 |  | 195 |  | 0.141 |  | 0.831 |  | 0.060 |  | 5.907 |  | 0.023 |  | 0.258 |  |
| AIMS\_mean\_NOTcollapsed |  | 1 |  | 198 |  | 2.456 |  | 0.810 |  | 0.058 |  | 0.330 |  | 2.342 |  | 2.569 |  |
|  |  | 2 |  | 195 |  | 2.614 |  | 0.766 |  | 0.055 |  | 0.293 |  | 2.506 |  | 2.722 |  |
| fscoresAIMS.Notcollapsed |  | 1 |  | 198 |  | -0.123 |  | 0.928 |  | 0.066 |  | -7.521 |  | -0.253 |  | 0.007 |  |
|  |  | 2 |  | 195 |  | 0.134 |  | 0.826 |  | 0.059 |  | 6.178 |  | 0.017 |  | 0.251 |  |
|  | | | | | | | | | | | | | | | | | |

### Descriptives Plots

#### AIMS\_mean\_collapsed

#### fscoresAIMS.collapsed

#### AIMS\_mean\_NOTcollapsed

#### fscoresAIMS.Notcollapsed
