# Supplementary figures and images for: Attitudes towards Italian Mafias Scale (AIMS): development and validation
Source: PeerJ. 2023 Oct 24;11:e16120. doi: 10.7717/peerj.16120 (PMC10607589; doi:10.7717/peerj.16120)

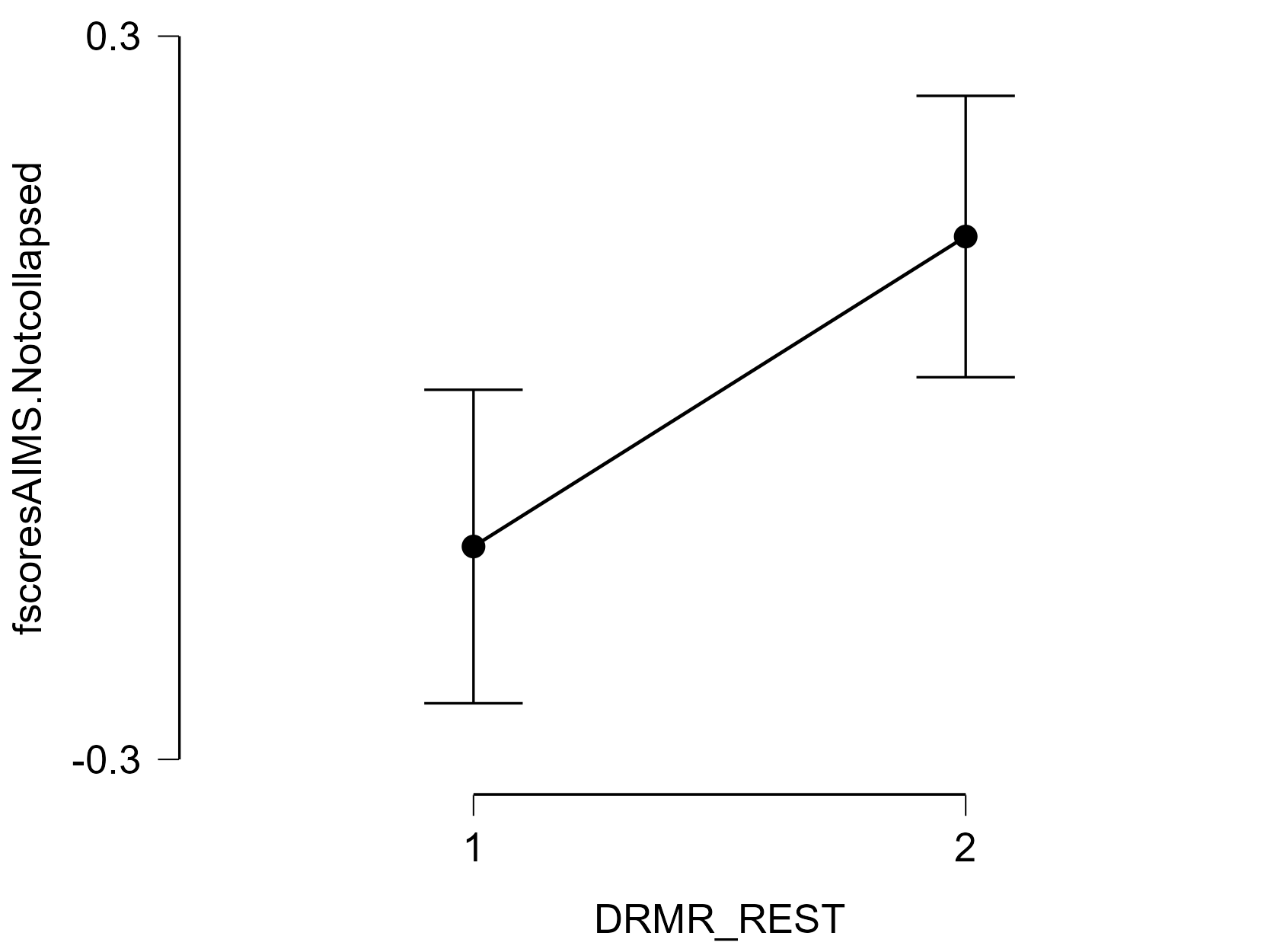

Supplement: Supplemental Information 4 — JASP (https://jasp-stats.org/) script to perform parametric, non-parametric analyses both for frequentist and bayesian approach for testing the difference between DRMR participants and those from the rest of Italy in Study 2 [file peerj-11-16120-s004.jasp › resources/1/_0_t-1514075659.png]

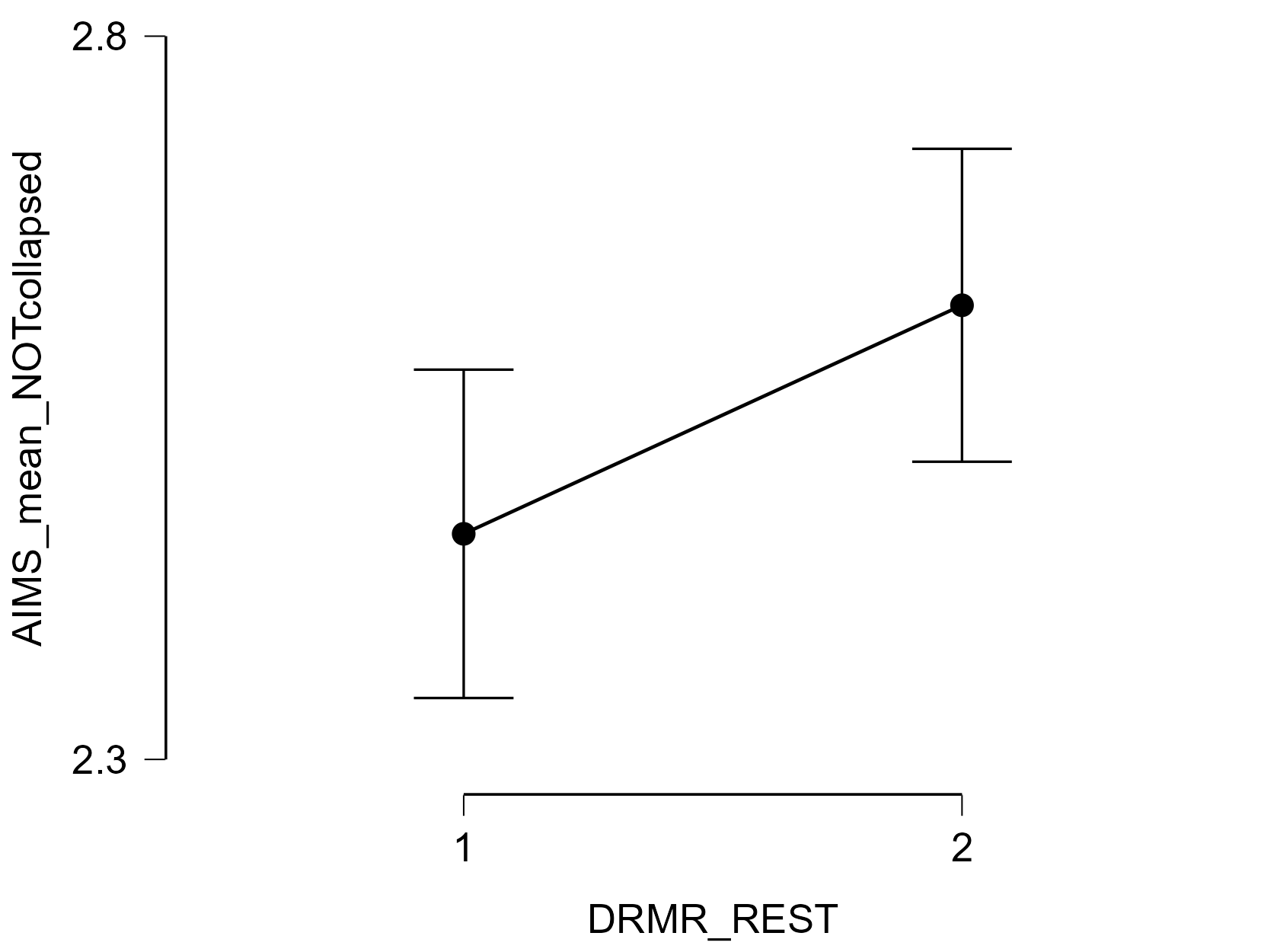

Supplement: Supplemental Information 4 — JASP (https://jasp-stats.org/) script to perform parametric, non-parametric analyses both for frequentist and bayesian approach for testing the difference between DRMR participants and those from the rest of Italy in Study 2 [file peerj-11-16120-s004.jasp › resources/1/_1_t-1514074080.png]

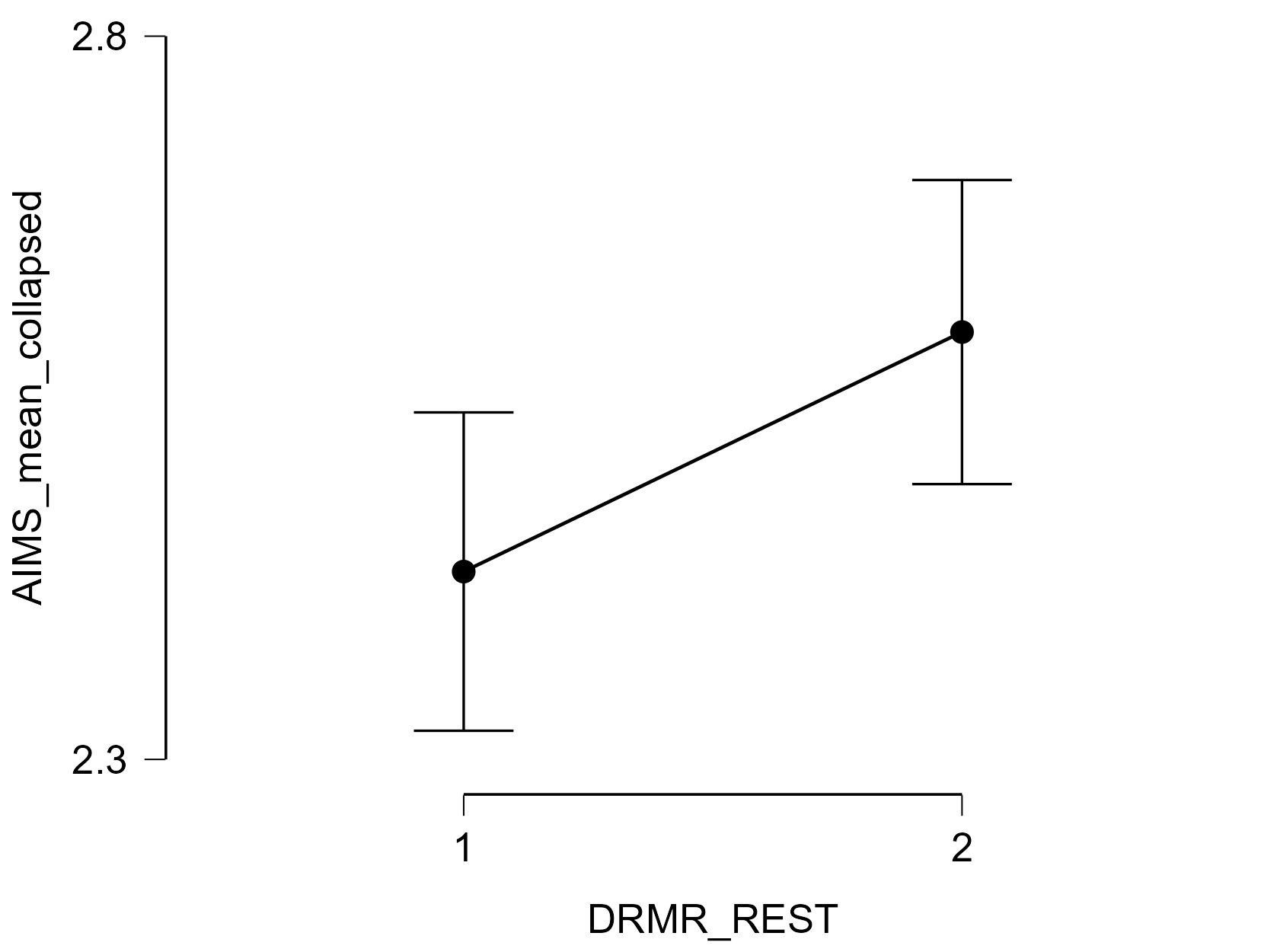

Supplement: Supplemental Information 4 — JASP (https://jasp-stats.org/) script to perform parametric, non-parametric analyses both for frequentist and bayesian approach for testing the difference between DRMR participants and those from the rest of Italy in Study 2 [file peerj-11-16120-s004.jasp › resources/1/_5_t-1585247383.png]

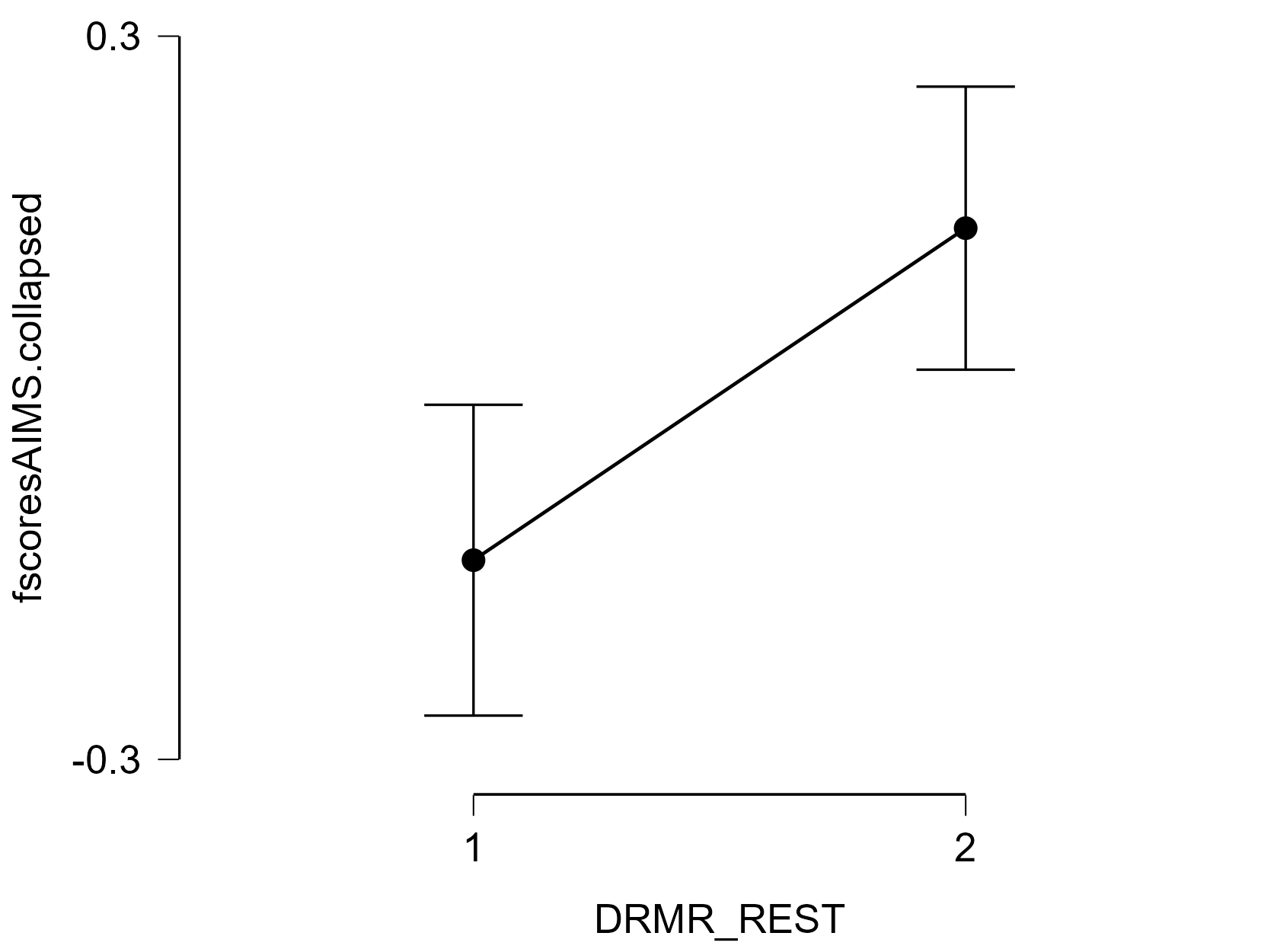

Supplement: Supplemental Information 4 — JASP (https://jasp-stats.org/) script to perform parametric, non-parametric analyses both for frequentist and bayesian approach for testing the difference between DRMR participants and those from the rest of Italy in Study 2 [file peerj-11-16120-s004.jasp › resources/1/_6_t-1585247027.png]

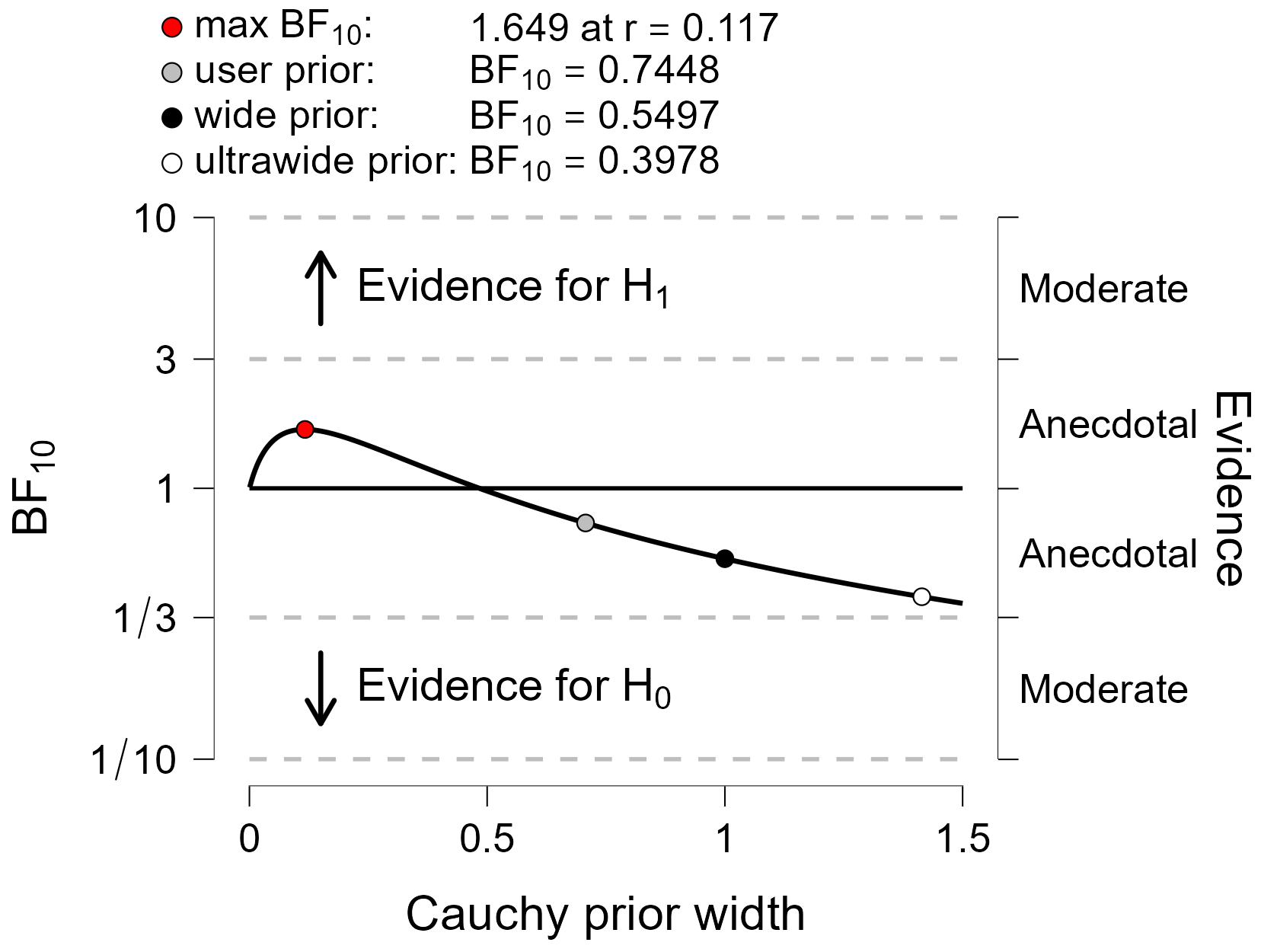

Supplement: Supplemental Information 4 — JASP (https://jasp-stats.org/) script to perform parametric, non-parametric analyses both for frequentist and bayesian approach for testing the difference between DRMR participants and those from the rest of Italy in Study 2 [file peerj-11-16120-s004.jasp › resources/2/_10_t-1491561052.png]

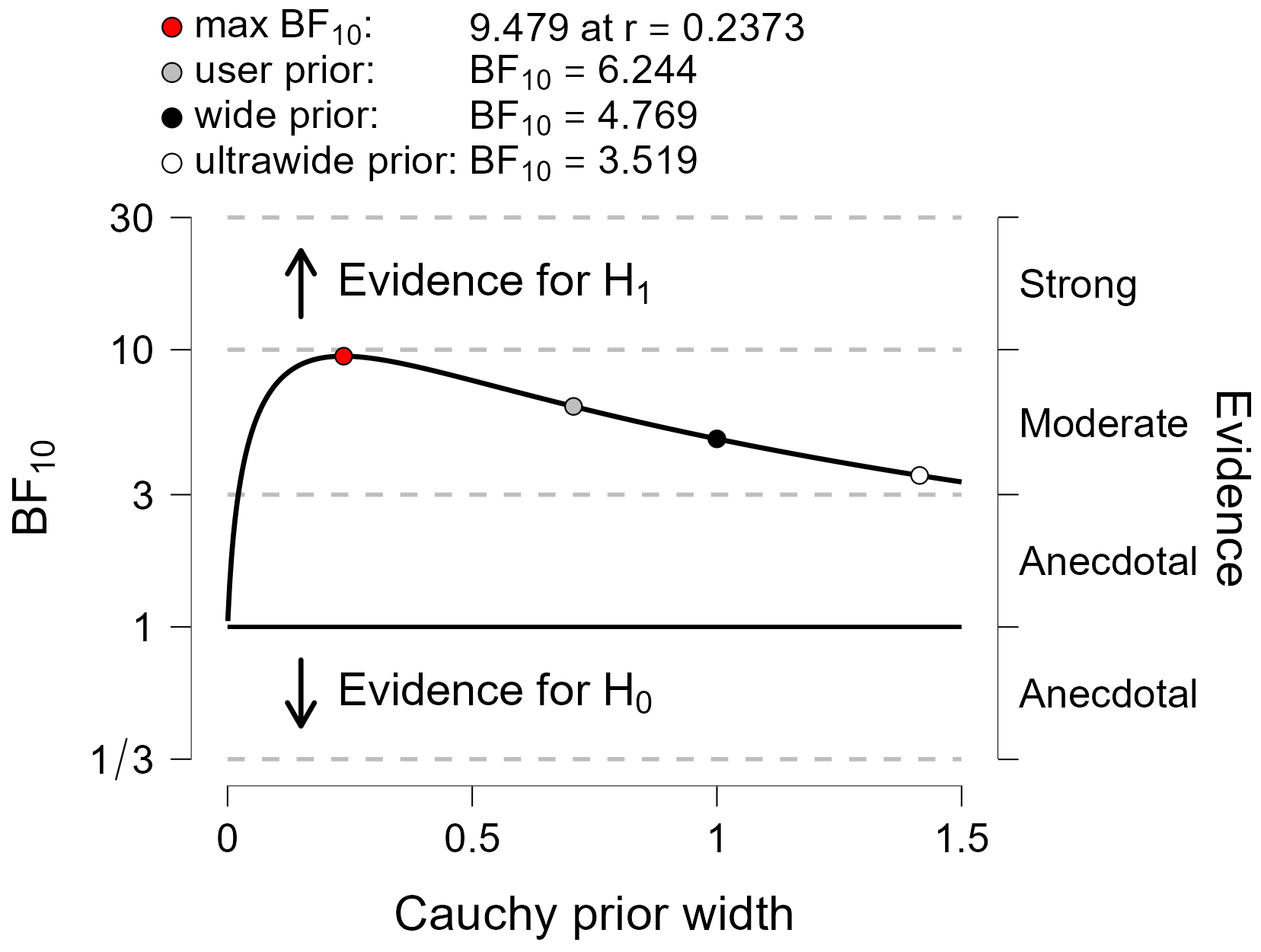

Supplement: Supplemental Information 4 — JASP (https://jasp-stats.org/) script to perform parametric, non-parametric analyses both for frequentist and bayesian approach for testing the difference between DRMR participants and those from the rest of Italy in Study 2 [file peerj-11-16120-s004.jasp › resources/2/_11_t-1491548048.png]

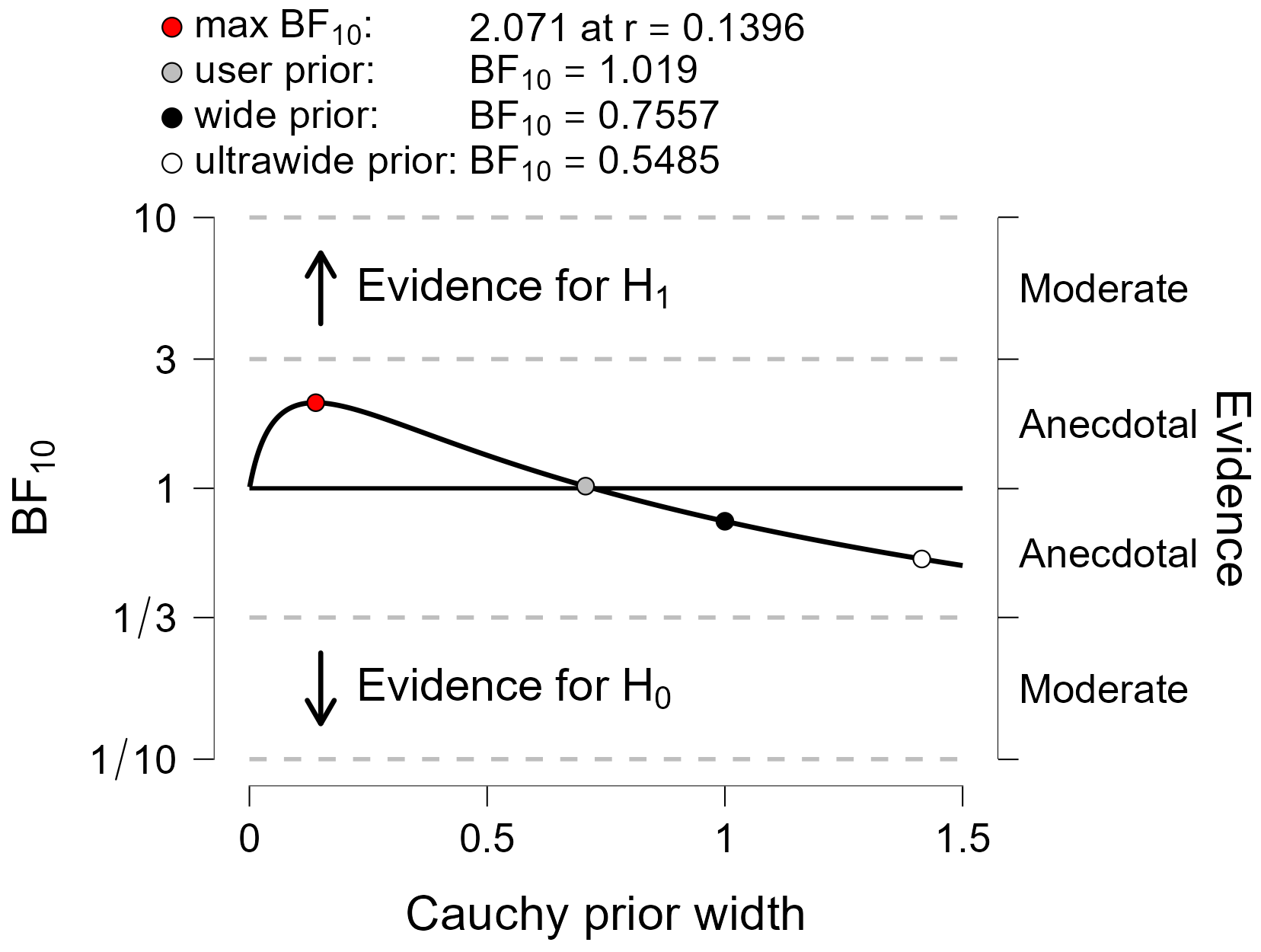

Supplement: Supplemental Information 4 — JASP (https://jasp-stats.org/) script to perform parametric, non-parametric analyses both for frequentist and bayesian approach for testing the difference between DRMR participants and those from the rest of Italy in Study 2 [file peerj-11-16120-s004.jasp › resources/2/_8_t-1491602344.png]

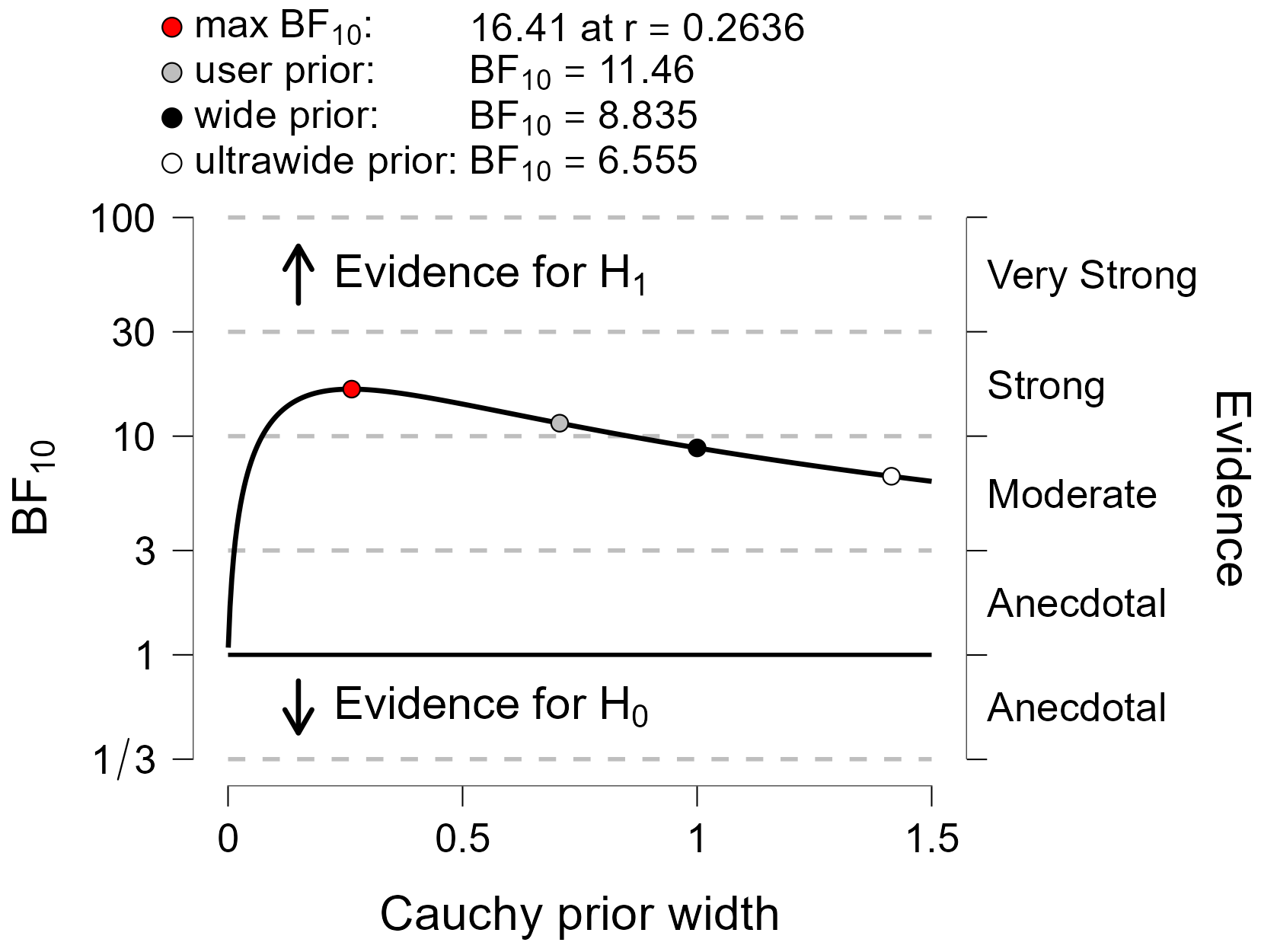

Supplement: Supplemental Information 4 — JASP (https://jasp-stats.org/) script to perform parametric, non-parametric analyses both for frequentist and bayesian approach for testing the difference between DRMR participants and those from the rest of Italy in Study 2 [file peerj-11-16120-s004.jasp › resources/2/_9_t-1491574523.png]
