# Supplementary material for: Attitudes towards Italian Mafias Scale (AIMS): development and validation
Source: PeerJ. 2023 Oct 24;11:e16120. doi: 10.7717/peerj.16120 (PMC10607589; doi:10.7717/peerj.16120)
Supplement: Supplemental Information 5 — Both final 18-items and complete list of the AIMS in Italian as presented in the original studies. [file peerj-11-16120-s005.docx]

**Italian version of the AIMS**

*AIMS complete list of items*

(E= Emotional component; B=Behavioral component; C=Cognitive component)

E1) Che tipo di emozioni [positive-negative] ti evoca la criminalità organizzata di stampo mafioso (Cosa Nostra/Camorra/’Ndrangheta/Sacra Corona Unita)?

E2) Quanto ti senti Sfavorevole/Favorevole rispetto alla criminalità organizzata di stampo mafioso (Cosa Nostra/Camorra/’Ndrangheta/Sacra Corona Unita)?

E3) Quanto ti senti Freddo/Caldo rispetto alla criminalità organizzata di stampo mafioso (Cosa Nostra/Camorra/’Ndrangheta/Sacra Corona Unita)?

E4) Quanto Disgusto ti evoca la criminalità organizzata di stampo mafioso (Cosa Nostra/Camorra/’Ndrangheta/Sacra Corona Unita)? R

E5) Quanta Tristezza ti evoca la criminalità organizzata di stampo mafioso (Cosa Nostra/Camorra/’Ndrangheta/Sacra Corona Unita)? R

E6) Quanta Gioia ti evoca la criminalità organizzata di stampo mafioso (Cosa Nostra/Camorra/’Ndrangheta/Sacra Corona Unita)?

E7) Quanta Rabbia ti evoca la criminalità organizzata di stampo mafioso (Cosa Nostra/Camorra/’Ndrangheta/Sacra Corona Unita)? R

E8) Quanto Disprezzo ti evoca la criminalità organizzata di stampo mafioso (Cosa Nostra/Camorra/’Ndrangheta/Sacra Corona Unita)? R

E9) Quanta Paura ti evoca la criminalità organizzata di stampo mafioso (Cosa Nostra/Camorra/’Ndrangheta/Sacra Corona Unita)? R

E10) Quanta Rassegnazione ti evoca la criminalità organizzata di stampo mafioso (Cosa Nostra/Camorra/’Ndrangheta/Sacra Corona Unita)? R

E11) Quanto Indifferenza ti evoca la criminalità organizzata di stampo mafioso (Cosa Nostra/Camorra/’Ndrangheta/Sacra Corona Unita)?”

E12) Quanta Ammirazione ti evoca la criminalità organizzata di stampo mafioso (Cosa Nostra/Camorra/’Ndrangheta/Sacra Corona Unita)?”

B1) Non avrei nulla in contrario se una persona appartenente alla criminalità organizzata di stampo mafioso (Cosa Nostra/Camorra/’Ndrangheta/Sacra Corona Unita) sposasse un membro della mia famiglia

B2) Non avrei nulla in contrario a sposare una persona che fa parte della criminalità organizzata di stampo mafioso (Cosa Nostra/Camorra/’Ndrangheta/Sacra Corona Unita)

B3) Denuncerei alle autorità competenti l’attività illecita di una persona appartenente alla criminalità organizzata di stampo mafioso (Cosa Nostra/Camorra/’Ndrangheta/Sacra Corona Unita), se ne fossi a conoscenza R

B4) Non mi ribellerei se una persona appartenente alla criminalità organizzata di stampo mafioso (Cosa Nostra/Camorra/’Ndrangheta/Sacra Corona Unita) mi chiedesse il pizzo

B5) Se ce ne fosse bisogno, sarei disposto a coprire le attività illecite di una persona appartenente alla criminalità organizzata di stampo mafioso (Cosa Nostra/Camorra/’Ndrangheta/Sacra Corona Unita)

B6) Interverrei fisicamente se vedessi una persona minacciata da individui appartenenti alla criminalità organizzata di stampo mafioso (Cosa Nostra/Camorra/’Ndrangheta/Sacra Corona Unita) R

B7) Se vedessi una persona minacciata da qualcuno appartenente alla criminalità organizzata di stampo mafioso (Cosa Nostra/Camorra/’Ndrangheta/Sacra Corona Unita), denuncerei il fatto alle autorità competenti

B8) Se una persona appartenente alla criminalità organizzata di stampo mafioso (Cosa Nostra/Camorra/’Ndrangheta/Sacra Corona Unita) mi offrisse di entrare in società con lei, accetterei

B9) Non accetterei mai di vivere come un boss della criminalità organizzata di stampo mafioso (Cosa Nostra/Camorra/’Ndrangheta/Sacra Corona Unita) R

B10) Non esiterei a chiedere l’aiuto di qualcuno appartenente alla criminalità organizzata di stampo mafioso (Cosa Nostra/Camorra/’Ndrangheta/Sacra Corona Unita) se dovessi farmi ridare dei soldi da qualcuno

B11) Sarei disposto a pagare una certa somma di denaro per avere la protezione della criminalità organizzata di stampo mafioso (Cosa Nostra/Camorra/’Ndrangheta/Sacra Corona Unita)

B12) Parteciperei a manifestazioni contro la criminalità organizzata di stampo mafioso (Cosa Nostra/Camorra/’Ndrangheta/Sacra Corona Unita) R

B13) Mi iscriverei a blog, associazioni o gruppi (social o che richiedono attività in presenza) contro la criminalità organizzata di stampo mafioso (Cosa Nostra/Camorra/’Ndrangheta/Sacra Corona Unita R

B14) Tollererei la presenza economica e fisica sul territorio della criminalità organizzata di stampo mafioso (Cosa Nostra/Camorra/’Ndrangheta/Sacra Corona Unita) purché sia nascosta

B15) Per me la criminalità organizzata di stampo mafioso (Cosa Nostra/Camorra/’Ndrangheta/Sacra Corona Unita) potrebbe continuare ad esistere purché non interferisca con la mia vita

B16) Collaborerei con le autorità competenti per far arrestare un membro della criminalità organizzata di stampo mafioso (Cosa Nostra/Camorra/’Ndrangheta/Sacra Corona Unita) R

B17) Non ci vedo nulla di male nell’usare l’intimidazione per riavere dei soldi

B18) Entrerei in contatto con la criminalità organizzata di stampo mafioso (Cosa Nostra/Camorra/’Ndrangheta/Sacra Corona Unita) per cercare lavoro

B19) Sarei disposto a investire del denaro per organizzare iniziative contro la criminalità organizzata di stampo mafioso (Cosa Nostra/Camorra/’Ndrangheta/Sacra Corona Unita) R

B20) Non denuncerei un mio parente se venissi a conoscenza che ha collaborato con la criminalità organizzata di stampo mafioso (Cosa Nostra/Camorra/’Ndrangheta/Sacra Corona Unita)

B21) Denuncerei l’attività illecita della criminalità organizzata di stampo mafioso (Cosa Nostra/Camorra/’Ndrangheta/Sacra Corona Unita) anche se questo vuol dire andare contro alle persone del mio vicinato e ai miei amici R

B22) Se una persona appartenente alla criminalità organizzata di stampo mafioso (Cosa Nostra/Camorra/’Ndrangheta/Sacra Corona Unita) mi offrisse dei soldi per favorirne attività illecite, accetterei

B23) Alle elezioni del mio comune di residenza non avrei problemi a votare per un candidato sostenuto dalla criminalità organizzata di stampo mafioso (Cosa Nostra/Camorra/’Ndrangheta/Sacra Corona Unita)

B24) Accetterei di votare per un candidato sostenuto dalla criminalità organizzata di stampo mafioso (Cosa Nostra/Camorra/’Ndrangheta/Sacra Corona Unita) se ricevessi dei favori in cambio

C1) Le persone appartenenti alla criminalità organizzata di stampo mafioso (Cosa Nostra/Camorra/’Ndrangheta/Sacra Corona Unita) sono meno oneste delle altre persone R

C2) La criminalità organizzata di stampo mafioso (Cosa Nostra/Camorra/’Ndrangheta/Sacra Corona Unita) contribuisce a creare posti di lavoro e ricchezza in Italia

C3) I membri della criminalità organizzata di stampo mafioso (Cosa Nostra/Camorra/’Ndrangheta/Sacra Corona Unita) sono persone perbene

C4) Le persone appartenenti alla criminalità organizzata di stampo mafioso (Cosa Nostra/Camorra/’Ndrangheta/Sacra Corona Unita) sono ingiustamente perseguitate dallo Stato

C5) Lo Stato non dovrebbe intromettersi nelle faide fra i clan della criminalità organizzata di stampo mafioso (Cosa Nostra/Camorra/’Ndrangheta/Sacra Corona Unita)

C6) Alcuni valori trasmessi dalle famiglie appartenenti alla criminalità organizzata di stampo mafioso (Cosa Nostra/Camorra/’Ndrangheta/Sacra Corona Unita) sono condivisibili

C7) In Italia ci sono altri problemi che dovrebbero avere la priorità rispetto a quello della criminalità organizzata di stampo mafioso (Cosa Nostra/Camorra/’Ndrangheta/Sacra Corona Unita)

C8) Essere affiliato alla criminalità di stampo mafioso (Cosa Nostra/Camorra/’Ndrangheta/Sacra Corona Unita) ti apre la strada verso la ricchezza

C9) Lavorare per la criminalità organizzata di stampo mafioso (Cosa Nostra/Camorra/’Ndrangheta/Sacra Corona Unita) è un lavoro come un altro

C10) Quella che chiamano criminalità organizzata di stampo mafioso (Cosa Nostra/Camorra/’Ndrangheta/Sacra Corona Unita) è in realtà criminalità comune

C11) E’ giusto pagare una somma di denaro per avere la protezione della criminalità organizzata di stampo mafioso (Cosa Nostra/Camorra/’Ndrangheta/Sacra Corona Unita)

C12) Il pizzo non è un'estorsione di denaro, bensi' la giusta ricompensa per ricevere la protezione da parte della criminalità organizzata di stampo mafioso (Cosa Nostra/'Ndrangheta/Camorra/Sacra Corona Unita)

C13) Le persone appartenenti alla criminalità di stampo mafioso (Cosa Nostra/Camorra/’Ndrangheta/Sacra Corona Unita) sono brave nelle loro attività

C14) Per una persona del Sud-Italia che voglia avere successo la criminalità organizzata di stampo mafioso (Cosa Nostra/Camorra/’Ndrangheta/Sacra Corona Unita) è l’unica soluzione

C15) La criminalità organizzata di stampo mafioso (Cosa Nostra/Camorra/’Ndrangheta/Sacra Corona Unita) impoverisce i territori in cui è presente R

C16) In fin dei conti la criminalità di stampo mafioso (Cosa Nostra/Camorra/’Ndrangheta/Sacra Corona Unita) aggira delle regole ingiuste

C17) Finché si uccidono fra di loro, le persone appartenenti alla criminalità organizzata di stampo mafioso (Cosa Nostra/Camorra/’Ndrangheta/Sacra Corona Unita) non sono un problema

C18) Non è giusto togliere la responsabilità genitoriale agli uomini appartenenti alla criminalità organizzata di stampo mafioso (Cosa Nostra/Camorra/’Ndrangheta/Sacra Corona Unita)

C19) Le scorte date alle persone minacciate dalla criminalità organizzata di stampo mafioso (Cosa Nostra/Camorra/’Ndrangheta/Sacra Corona Unita) sono solo uno spreco di soldi per lo Stato

C20) I problemi legati alla criminalità organizzata di stampo mafioso (Cosa Nostra/Camorra/’Ndrangheta/Sacra Corona Unita) sono molto ingigantiti

C21) Le persone appartenenti alla criminalità organizzata di stampo mafioso (Cosa Nostra/Camorra/’Ndrangheta/Sacra Corona Unita) hanno ragione quando dicono che i pentiti sono dei traditori

C22) La criminalità organizzata di stampo mafioso (Cosa Nostra/Camorra/’Ndrangheta/Sacra Corona Unita) insegna valori importanti quali onore e rispetto

C23) Le persone appartenenti alla criminalità organizzata di stampo mafioso (Cosa Nostra/Camorra/’Ndrangheta/Sacra Corona Unita) fanno solo ciò che è giusto per il proprio clan famigliare

C24) Se lo Stato cercasse un dialogo con la criminalità organizzata di stampo mafioso (Cosa Nostra/Camorra/’Ndrangheta/Sacra Corona Unita), non ci sarebbe tutta questa violenza

C25) Affiliarsi alla criminalità organizzata di stampo mafioso (Cosa Nostra/Camorra/’Ndrangheta/Sacra Corona Unita) è un modo veloce e facile di ottenere tanti soldi

C26) Trovo giusto confiscare i beni alle persone appartenenti alla criminalità organizzata di stampo mafioso (Cosa Nostra/Camorra/’Ndrangheta/Sacra Corona Unita) R

C27) L’importante è che la criminalità organizzata di stampo mafioso (Cosa Nostra/Camorra/’Ndrangheta/Sacra Corona Unita) non crei problemi a me

C28) Bisognerebbe dare l’opportunità alla criminalità organizzata di stampo mafioso (Cosa Nostra/Camorra/’Ndrangheta/Sacra Corona Unita) di legalizzarsi

C29) Un membro della criminalità organizzata di stampo mafioso (Cosa Nostra/Camorra/’Ndrangheta/Sacra Corona Unita) è una persona più furba delle altre

C30) Le persone appartenenti alla criminalità organizzata di stampo mafioso (Cosa Nostra/Camorra/’Ndrangheta/Sacra Corona Unita) pensano solo al proprio interesse R

C31) Le persone appartenenti alla criminalità organizzata di stampo mafioso (Cosa Nostra/Camorra/’Ndrangheta/Sacra Corona Unita) meritano le disgrazie che capitano loro R

C32) La criminalità organizzata di stampo mafioso (Cosa Nostra/Camorra/’Ndrangheta/Sacra Corona Unita) non può essere sconfitta

C33) La criminalità organizzata di stampo mafioso (Cosa Nostra/Camorra/’Ndrangheta/Sacra Corona Unita) è parte integrante della cultura italiana

C34) Le persone appartenenti alla criminalità organizzata di stampo mafioso (Cosa Nostra/Camorra/’Ndrangheta/Sacra Corona Unita) sono persone che sanno farsi rispettare

C35) La criminalità organizzata di stampo mafioso (Cosa Nostra/Camorra/’Ndrangheta/Sacra Corona Unita) offre opportunità lavorative che lo Stato non è in grado di dare

C36) Lo Stato dovrebbe impegnare più risorse nel combattere la criminalità organizzata di stampo mafioso (Cosa Nostra/Camorra/’Ndrangheta/Sacra Corona Unita) R

C37) Lo Stato dovrebbe occuparsi della corruzione dei suoi politici invece che pensare alla criminalità organizzata di stampo mafioso (Cosa Nostra/Camorra/’Ndrangheta/Sacra Corona Unita)

C38) Le persone che assistono alle attività illecite della criminalità organizzata di stampo mafioso (Cosa Nostra/Camorra/’Ndrangheta/Sacra Corona Unita) dovrebbero farsi gli affari loro e non denunciare alle autorità competenti

C39) La criminalità organizzata di stampo mafioso (Cosa Nostra/Camorra/’Ndrangheta/Sacra Corona Unita) è la giusta risposta all’abbandono del Sud-Italia da parte dello Stato

C40) La criminalità organizzata di stampo mafioso (Cosa Nostra/Camorra/’Ndrangheta/Sacra Corona Unita) è un problema solo delle regioni del Sud-Italia

*R = reversed score

***Final 18-items AIMS***

E5) Quanta Tristezza ti evoca la criminalità organizzata di stampo mafioso (Cosa Nostra/Camorra/’Ndrangheta/Sacra Corona Unita)? R

E7) Quanta Rabbia ti evoca la criminalità organizzata di stampo mafioso (Cosa Nostra/Camorra/’Ndrangheta/Sacra Corona Unita)? R

E11) Quanta Indifferenza ti evoca la criminalità organizzata di stampo mafioso (Cosa Nostra/Camorra/’Ndrangheta/Sacra Corona Unita)?

B3) Denuncerei alle autorità competenti l’attività illecita di una persona appartenente alla criminalità organizzata di stampo mafioso (Cosa Nostra/Camorra/’Ndrangheta/Sacra Corona Unita), se ne fossi a conoscenza R

B4) Non mi ribellerei se una persona appartenente alla criminalità organizzata di stampo mafioso (Cosa Nostra/Camorra/’Ndrangheta/Sacra Corona Unita) mi chiedesse il pizzo

B7) Se vedessi una persona minacciata da qualcuno appartenente alla criminalità organizzata di stampo mafioso (Cosa Nostra/Camorra/’Ndrangheta/Sacra Corona Unita), denuncerei il fatto alle autorità competenti

B12) Parteciperei a manifestazioni contro la criminalità organizzata di stampo mafioso (Cosa Nostra/Camorra/’Ndrangheta/Sacra Corona Unita) R

B19) Sarei disposto a investire del denaro per organizzare iniziative contro la criminalità organizzata di stampo mafioso (Cosa Nostra/Camorra/’Ndrangheta/Sacra Corona Unita) R

B20) Non denuncerei un mio parente se venissi a conoscenza che ha collaborato con la criminalità organizzata di stampo mafioso (Cosa Nostra/Camorra/’Ndrangheta/Sacra Corona Unita)

B21) Denuncerei l’attività illecita della criminalità organizzata di stampo mafioso (Cosa Nostra/Camorra/’Ndrangheta/Sacra Corona Unita) anche se questo vuol dire andare contro alle persone del mio vicinato e ai miei amici R

C1) Le persone appartenenti alla criminalità organizzata di stampo mafioso (Cosa Nostra/Camorra/’Ndrangheta/Sacra Corona Unita) sono meno oneste delle altre persone R

C6) Alcuni valori trasmessi dalle famiglie appartenenti alla criminalità organizzata di stampo mafioso (Cosa Nostra/Camorra/’Ndrangheta/Sacra Corona Unita) sono condivisibili

C13) Le persone appartenenti alla criminalità di stampo mafioso (Cosa Nostra/Camorra/’Ndrangheta/Sacra Corona Unita) sono brave nelle loro attività

C15) La criminalità organizzata di stampo mafioso (Cosa Nostra/Camorra/’Ndrangheta/Sacra Corona Unita) impoverisce i territori in cui è presente R

C29) Un membro della criminalità organizzata di stampo mafioso (Cosa Nostra/Camorra/’Ndrangheta/Sacra Corona Unita) è una persona più furba delle altre

C30) Le persone appartenenti alla criminalità organizzata di stampo mafioso (Cosa Nostra/Camorra/’Ndrangheta/Sacra Corona Unita) pensano solo al proprio interesse R

C34) Le persone appartenenti alla criminalità organizzata di stampo mafioso (Cosa Nostra/Camorra/’Ndrangheta/Sacra Corona Unita) sono persone che sanno farsi rispettare

C35) La criminalità organizzata di stampo mafioso (Cosa Nostra/Camorra/’Ndrangheta/Sacra Corona Unita) offre opportunità lavorative che lo Stato non è in grado di dare

*R = reversed score
