# Supplementary material for: Attitudes towards Italian Mafias Scale (AIMS): development and validation
Source: PeerJ. 2023 Oct 24;11:e16120. doi: 10.7717/peerj.16120 (PMC10607589; doi:10.7717/peerj.16120)
Supplement: Supplemental Information 6 — Complete and final versions, both in English and Italian, of the AIMS; Supplementary analyses, with bayesian and frequentist approaches, with 7 response categories and with AIMS factor scores. [file peerj-11-16120-s006.docx]

**Supplementary materials**

***AIMS complete list of items (English)***

(E= Emotional component; B=Behavioral component; C=Cognitive component)

E1) What kind of emotions [positive-negative] does mafia organized crime evoke in you?

E2) How much favorable/unfavorable do you feel towards mafia organized crime?

E3) How much cold/hot do you feel towards mafia organized crime?

E4) How much disgust mafia organized crime evoke in you? R

E5) How much sadness mafia organized crime evoke in you? R

E6) How much joy mafia organized crime evoke in you?

E7) How much anger mafia organized crime evoke in you? R

E8) How much contempt mafia organized crime evoke in you? R

E9) How much fear mafia organized crime evoke in you? R

E10) How much resignation mafia organized crime evoke in you? R

E11) How much indifference mafia organized crime evoke in you?

E12) How much admiration mafia organized crime evoke in you?

B1) I would have nothing against a member of mafia organized crime marrying one of my family

B2) I would have nothing against marrying a member of mafia organized crime

B3) I would report to the competent authorities the illegal activities committed by a member of mafia organized crime, if I knew about them R

B4) I would not rebel against a member of mafia organized crime making an extortion to me

B5) If needed, I would cover for the illegal activities of a member of mafia organized crime

B6) I would physically intervene, if I saw someone threatened by members of mafia organized crime R

B7) If I saw a person threatened by a member of mafia organized crime, I would report it to the competent authorities R

B8) If a member of mafia organized crime offered me to go into business with him, I would accept

B9) I would never accept to live like a boss of mafia organized crime R

B10) I would not hesitate to ask for the help of a member of mafia organized crime, if I had to get my money back from someone

B11) I would pay some money to receive protection from mafia organized crime

B12) I would participate in events against mafia organized crime R

B13) I would register to blog, associations, groups (on the internet or in presence) against mafia organized crime R

B14) I would tolerate the economic and physical presence of mafia organized crime on my territory if hidden

B15) To me, mafia organized crime could continue existing as long as it does not interfere with my life

B16) I would collaborate with the competent authorities to arrest a member of mafia organized crime R

B17) There is nothing wrong in using intimidation to get some money back

B18) I would get in touch with mafia organized crime to get a job

B19) I would invest my money to organize initiatives against mafia organized crime R

B20) I would not report a relative of mine if I knew that he/she collaborated with mafia organized crime

B21) I would report the illegal activities of mafia organized crime, even if that would mean going against my neighbours and friends R

B22) If a member of organized crime offered me some money to favor his illegal activities, I would accept

B23) I would have no problems to vote for a candidate supported by mafia organized crime in my hometown elections

B24) I would accept to vote for a candidate supported by mafia organized crime, If I get some favors back

C1) Members of mafia organized crime are less honest than other people R

C2) Mafia organized crime contributes to create jobs and richness in Italy

C3) Members of mafia organized crime are good people

C4) Members of mafia organized crime are unfairly persecuted by the State

C5) The State should not intrude in the feuds between clans of mafia organized crime

C6) Some values transmitted by mafia organized crime clans are agreeable

C7) There are other problems in Italy that should have priority over mafia organized crime

C8) Being affiliated to mafia organized crime pave your way to richness

C9) Working for mafia organized crime is a job like any other

C10) What it is called mafia organized crime is actually common crime

C11) It is acceptable paying some money to mafia organized crime to receive its protection

C12) “Pizzo” is not an extorsion, rather the right price to receive protection from mafia organized crime

C13) Members of organized crime are good at their activities

C14) For a person of Southern-Italy that wants to succeed in life mafia organized crime is the only solution

C15) Mafia organized crime impoverishes the territories where it is present R

C16) After all, mafia organized crime bypasses unfair rules

C17) As long as they kill each others, members of mafia organized crime are not a problem

C18) It is not fair taking away parental authority from members of mafia organized crime

C19) The guards assigned to people threatened by mafia organized crime are just a waste of money for the State

C20) Problems related to mafia organized crime are very much exagerated

C21) Members of mafia organized crime are right when they say that repentant mafiosi are traitors

C22) Mafia organized crime teaches important values such as honor and respect

C23) Members of mafia organized crime just do what is right for their clan

C24) If the State looked for a dialogue with mafia organized crime, there would not be all this violence

C25) Affiliating to mafia organized crime is a way to easily and quickly get a lot of money

C26) I think it is right to confiscate the goods of mafia organized crime R

C27) The important thing is that mafia organzized crime would not cause troubles to me

C28) We should give mafia organized crime the chance to make its position legal

C29) A member of mafia organized crime is smarter than other people

C30) Members of mafia organized crime think only about their own interest R

C31) Members of mafia organized crime deserve the bad things that happen to them R

C32) Mafia organized crime cannot be defeated

C33) Mafia organized crime is part and parcel of Italian culture

C34) Members of mafia organized crime know how to be respected

C35) Mafia organized crime offers you job opportunities that the State is not able to give

C36) The State should invest more resources to fight mafia organized crime R

C37) The State should deal with its corrupted politicians instead of thinking about mafia organized crime

C38) People witnessing the illegal activities of mafia organized crime should mind their own business and not report them to the competent authorities

C39) Mafia organized crime is the right answer to abandonment of Southern-Italy by the State

C40) Mafia organized crime is a problem only for Southern-Italy

*R = reversed score

***AIMS complete list of items (Italian)***

E1) Che tipo di emozioni [positive-negative] ti evoca la criminalità organizzata di stampo mafioso (Cosa Nostra/Camorra/’Ndrangheta/Sacra Corona Unita)?

E2) Quanto ti senti Sfavorevole/Favorevole rispetto alla criminalità organizzata di stampo mafioso (Cosa Nostra/Camorra/’Ndrangheta/Sacra Corona Unita)?

E3) Quanto ti senti Freddo/Caldo rispetto alla criminalità organizzata di stampo mafioso (Cosa Nostra/Camorra/’Ndrangheta/Sacra Corona Unita)?

E4) Quanto Disgusto ti evoca la criminalità organizzata di stampo mafioso (Cosa Nostra/Camorra/’Ndrangheta/Sacra Corona Unita)? R

E5) Quanta Tristezza ti evoca la criminalità organizzata di stampo mafioso (Cosa Nostra/Camorra/’Ndrangheta/Sacra Corona Unita)? R

E6) Quanta Gioia ti evoca la criminalità organizzata di stampo mafioso (Cosa Nostra/Camorra/’Ndrangheta/Sacra Corona Unita)?

E7) Quanta Rabbia ti evoca la criminalità organizzata di stampo mafioso (Cosa Nostra/Camorra/’Ndrangheta/Sacra Corona Unita)? R

E8) Quanto Disprezzo ti evoca la criminalità organizzata di stampo mafioso (Cosa Nostra/Camorra/’Ndrangheta/Sacra Corona Unita)? R

E9) Quanta Paura ti evoca la criminalità organizzata di stampo mafioso (Cosa Nostra/Camorra/’Ndrangheta/Sacra Corona Unita)? R

E10) Quanta Rassegnazione ti evoca la criminalità organizzata di stampo mafioso (Cosa Nostra/Camorra/’Ndrangheta/Sacra Corona Unita)? R

E11) Quanto Indifferenza ti evoca la criminalità organizzata di stampo mafioso (Cosa Nostra/Camorra/’Ndrangheta/Sacra Corona Unita)?”

E12) Quanta Ammirazione ti evoca la criminalità organizzata di stampo mafioso (Cosa Nostra/Camorra/’Ndrangheta/Sacra Corona Unita)?”

B1) Non avrei nulla in contrario se una persona appartenente alla criminalità organizzata di stampo mafioso (Cosa Nostra/Camorra/’Ndrangheta/Sacra Corona Unita) sposasse un membro della mia famiglia

B2) Non avrei nulla in contrario a sposare una persona che fa parte della criminalità organizzata di stampo mafioso (Cosa Nostra/Camorra/’Ndrangheta/Sacra Corona Unita)

B3) Denuncerei alle autorità competenti l’attività illecita di una persona appartenente alla criminalità organizzata di stampo mafioso (Cosa Nostra/Camorra/’Ndrangheta/Sacra Corona Unita), se ne fossi a conoscenza R

B4) Non mi ribellerei se una persona appartenente alla criminalità organizzata di stampo mafioso (Cosa Nostra/Camorra/’Ndrangheta/Sacra Corona Unita) mi chiedesse il pizzo

B5) Se ce ne fosse bisogno, sarei disposto a coprire le attività illecite di una persona appartenente alla criminalità organizzata di stampo mafioso (Cosa Nostra/Camorra/’Ndrangheta/Sacra Corona Unita)

B6) Interverrei fisicamente se vedessi una persona minacciata da individui appartenenti alla criminalità organizzata di stampo mafioso (Cosa Nostra/Camorra/’Ndrangheta/Sacra Corona Unita) R

B7) Se vedessi una persona minacciata da qualcuno appartenente alla criminalità organizzata di stampo mafioso (Cosa Nostra/Camorra/’Ndrangheta/Sacra Corona Unita), denuncerei il fatto alle autorità competenti

B8) Se una persona appartenente alla criminalità organizzata di stampo mafioso (Cosa Nostra/Camorra/’Ndrangheta/Sacra Corona Unita) mi offrisse di entrare in società con lei, accetterei

B9) Non accetterei mai di vivere come un boss della criminalità organizzata di stampo mafioso (Cosa Nostra/Camorra/’Ndrangheta/Sacra Corona Unita) R

B10) Non esiterei a chiedere l’aiuto di qualcuno appartenente alla criminalità organizzata di stampo mafioso (Cosa Nostra/Camorra/’Ndrangheta/Sacra Corona Unita) se dovessi farmi ridare dei soldi da qualcuno

B11) Sarei disposto a pagare una certa somma di denaro per avere la protezione della criminalità organizzata di stampo mafioso (Cosa Nostra/Camorra/’Ndrangheta/Sacra Corona Unita)

B12) Parteciperei a manifestazioni contro la criminalità organizzata di stampo mafioso (Cosa Nostra/Camorra/’Ndrangheta/Sacra Corona Unita) R

B13) Mi iscriverei a blog, associazioni o gruppi (social o che richiedono attività in presenza) contro la criminalità organizzata di stampo mafioso (Cosa Nostra/Camorra/’Ndrangheta/Sacra Corona Unita R

B14) Tollererei la presenza economica e fisica sul territorio della criminalità organizzata di stampo mafioso (Cosa Nostra/Camorra/’Ndrangheta/Sacra Corona Unita) purché sia nascosta

B15) Per me la criminalità organizzata di stampo mafioso (Cosa Nostra/Camorra/’Ndrangheta/Sacra Corona Unita) potrebbe continuare ad esistere purché non interferisca con la mia vita

B16) Collaborerei con le autorità competenti per far arrestare un membro della criminalità organizzata di stampo mafioso (Cosa Nostra/Camorra/’Ndrangheta/Sacra Corona Unita) R

B17) Non ci vedo nulla di male nell’usare l’intimidazione per riavere dei soldi

B18) Entrerei in contatto con la criminalità organizzata di stampo mafioso (Cosa Nostra/Camorra/’Ndrangheta/Sacra Corona Unita) per cercare lavoro

B19) Sarei disposto a investire del denaro per organizzare iniziative contro la criminalità organizzata di stampo mafioso (Cosa Nostra/Camorra/’Ndrangheta/Sacra Corona Unita) R

B20) Non denuncerei un mio parente se venissi a conoscenza che ha collaborato con la criminalità organizzata di stampo mafioso (Cosa Nostra/Camorra/’Ndrangheta/Sacra Corona Unita)

B21) Denuncerei l’attività illecita della criminalità organizzata di stampo mafioso (Cosa Nostra/Camorra/’Ndrangheta/Sacra Corona Unita) anche se questo vuol dire andare contro alle persone del mio vicinato e ai miei amici R

B22) Se una persona appartenente alla criminalità organizzata di stampo mafioso (Cosa Nostra/Camorra/’Ndrangheta/Sacra Corona Unita) mi offrisse dei soldi per favorirne attività illecite, accetterei

B23) Alle elezioni del mio comune di residenza non avrei problemi a votare per un candidato sostenuto dalla criminalità organizzata di stampo mafioso (Cosa Nostra/Camorra/’Ndrangheta/Sacra Corona Unita)

B24) Accetterei di votare per un candidato sostenuto dalla criminalità organizzata di stampo mafioso (Cosa Nostra/Camorra/’Ndrangheta/Sacra Corona Unita) se ricevessi dei favori in cambio

C1) Le persone appartenenti alla criminalità organizzata di stampo mafioso (Cosa Nostra/Camorra/’Ndrangheta/Sacra Corona Unita) sono meno oneste delle altre persone R

C2) La criminalità organizzata di stampo mafioso (Cosa Nostra/Camorra/’Ndrangheta/Sacra Corona Unita) contribuisce a creare posti di lavoro e ricchezza in Italia

C3) I membri della criminalità organizzata di stampo mafioso (Cosa Nostra/Camorra/’Ndrangheta/Sacra Corona Unita) sono persone perbene

C4) Le persone appartenenti alla criminalità organizzata di stampo mafioso (Cosa Nostra/Camorra/’Ndrangheta/Sacra Corona Unita) sono ingiustamente perseguitate dallo Stato

C5) Lo Stato non dovrebbe intromettersi nelle faide fra i clan della criminalità organizzata di stampo mafioso (Cosa Nostra/Camorra/’Ndrangheta/Sacra Corona Unita)

C6) Alcuni valori trasmessi dalle famiglie appartenenti alla criminalità organizzata di stampo mafioso (Cosa Nostra/Camorra/’Ndrangheta/Sacra Corona Unita) sono condivisibili

C7) In Italia ci sono altri problemi che dovrebbero avere la priorità rispetto a quello della criminalità organizzata di stampo mafioso (Cosa Nostra/Camorra/’Ndrangheta/Sacra Corona Unita)

C8) Essere affiliato alla criminalità di stampo mafioso (Cosa Nostra/Camorra/’Ndrangheta/Sacra Corona Unita) ti apre la strada verso la ricchezza

C9) Lavorare per la criminalità organizzata di stampo mafioso (Cosa Nostra/Camorra/’Ndrangheta/Sacra Corona Unita) è un lavoro come un altro

C10) Quella che chiamano criminalità organizzata di stampo mafioso (Cosa Nostra/Camorra/’Ndrangheta/Sacra Corona Unita) è in realtà criminalità comune

C11) E’ giusto pagare una somma di denaro per avere la protezione della criminalità organizzata di stampo mafioso (Cosa Nostra/Camorra/’Ndrangheta/Sacra Corona Unita)

C12) Il pizzo non è un'estorsione di denaro, bensi' la giusta ricompensa per ricevere la protezione da parte della criminalità organizzata di stampo mafioso (Cosa Nostra/'Ndrangheta/Camorra/Sacra Corona Unita)

C13) Le persone appartenenti alla criminalità di stampo mafioso (Cosa Nostra/Camorra/’Ndrangheta/Sacra Corona Unita) sono brave nelle loro attività

C14) Per una persona del Sud-Italia che voglia avere successo la criminalità organizzata di stampo mafioso (Cosa Nostra/Camorra/’Ndrangheta/Sacra Corona Unita) è l’unica soluzione

C15) La criminalità organizzata di stampo mafioso (Cosa Nostra/Camorra/’Ndrangheta/Sacra Corona Unita) impoverisce i territori in cui è presente R

C16) In fin dei conti la criminalità di stampo mafioso (Cosa Nostra/Camorra/’Ndrangheta/Sacra Corona Unita) aggira delle regole ingiuste

C17) Finché si uccidono fra di loro, le persone appartenenti alla criminalità organizzata di stampo mafioso (Cosa Nostra/Camorra/’Ndrangheta/Sacra Corona Unita) non sono un problema

C18) Non è giusto togliere la responsabilità genitoriale agli uomini appartenenti alla criminalità organizzata di stampo mafioso (Cosa Nostra/Camorra/’Ndrangheta/Sacra Corona Unita)

C19) Le scorte date alle persone minacciate dalla criminalità organizzata di stampo mafioso (Cosa Nostra/Camorra/’Ndrangheta/Sacra Corona Unita) sono solo uno spreco di soldi per lo Stato

C20) I problemi legati alla criminalità organizzata di stampo mafioso (Cosa Nostra/Camorra/’Ndrangheta/Sacra Corona Unita) sono molto ingigantiti

C21) Le persone appartenenti alla criminalità organizzata di stampo mafioso (Cosa Nostra/Camorra/’Ndrangheta/Sacra Corona Unita) hanno ragione quando dicono che i pentiti sono dei traditori

C22) La criminalità organizzata di stampo mafioso (Cosa Nostra/Camorra/’Ndrangheta/Sacra Corona Unita) insegna valori importanti quali onore e rispetto

C23) Le persone appartenenti alla criminalità organizzata di stampo mafioso (Cosa Nostra/Camorra/’Ndrangheta/Sacra Corona Unita) fanno solo ciò che è giusto per il proprio clan famigliare

C24) Se lo Stato cercasse un dialogo con la criminalità organizzata di stampo mafioso (Cosa Nostra/Camorra/’Ndrangheta/Sacra Corona Unita), non ci sarebbe tutta questa violenza

C25) Affiliarsi alla criminalità organizzata di stampo mafioso (Cosa Nostra/Camorra/’Ndrangheta/Sacra Corona Unita) è un modo veloce e facile di ottenere tanti soldi

C26) Trovo giusto confiscare i beni alle persone appartenenti alla criminalità organizzata di stampo mafioso (Cosa Nostra/Camorra/’Ndrangheta/Sacra Corona Unita) R

C27) L’importante è che la criminalità organizzata di stampo mafioso (Cosa Nostra/Camorra/’Ndrangheta/Sacra Corona Unita) non crei problemi a me

C28) Bisognerebbe dare l’opportunità alla criminalità organizzata di stampo mafioso (Cosa Nostra/Camorra/’Ndrangheta/Sacra Corona Unita) di legalizzarsi

C29) Un membro della criminalità organizzata di stampo mafioso (Cosa Nostra/Camorra/’Ndrangheta/Sacra Corona Unita) è una persona più furba delle altre

C30) Le persone appartenenti alla criminalità organizzata di stampo mafioso (Cosa Nostra/Camorra/’Ndrangheta/Sacra Corona Unita) pensano solo al proprio interesse R

C31) Le persone appartenenti alla criminalità organizzata di stampo mafioso (Cosa Nostra/Camorra/’Ndrangheta/Sacra Corona Unita) meritano le disgrazie che capitano loro R

C32) La criminalità organizzata di stampo mafioso (Cosa Nostra/Camorra/’Ndrangheta/Sacra Corona Unita) non può essere sconfitta

C33) La criminalità organizzata di stampo mafioso (Cosa Nostra/Camorra/’Ndrangheta/Sacra Corona Unita) è parte integrante della cultura italiana

C34) Le persone appartenenti alla criminalità organizzata di stampo mafioso (Cosa Nostra/Camorra/’Ndrangheta/Sacra Corona Unita) sono persone che sanno farsi rispettare

C35) La criminalità organizzata di stampo mafioso (Cosa Nostra/Camorra/’Ndrangheta/Sacra Corona Unita) offre opportunità lavorative che lo Stato non è in grado di dare

C36) Lo Stato dovrebbe impegnare più risorse nel combattere la criminalità organizzata di stampo mafioso (Cosa Nostra/Camorra/’Ndrangheta/Sacra Corona Unita) R

C37) Lo Stato dovrebbe occuparsi della corruzione dei suoi politici invece che pensare alla criminalità organizzata di stampo mafioso (Cosa Nostra/Camorra/’Ndrangheta/Sacra Corona Unita)

C38) Le persone che assistono alle attività illecite della criminalità organizzata di stampo mafioso (Cosa Nostra/Camorra/’Ndrangheta/Sacra Corona Unita) dovrebbero farsi gli affari loro e non denunciare alle autorità competenti

C39) La criminalità organizzata di stampo mafioso (Cosa Nostra/Camorra/’Ndrangheta/Sacra Corona Unita) è la giusta risposta all’abbandono del Sud-Italia da parte dello Stato

C40) La criminalità organizzata di stampo mafioso (Cosa Nostra/Camorra/’Ndrangheta/Sacra Corona Unita) è un problema solo delle regioni del Sud-Italia

*R = reversed score

***Final 18-items AIMS (Italian)***

E5) Quanta Tristezza ti evoca la criminalità organizzata di stampo mafioso (Cosa Nostra/Camorra/’Ndrangheta/Sacra Corona Unita)? R

E7) Quanta Rabbia ti evoca la criminalità organizzata di stampo mafioso (Cosa Nostra/Camorra/’Ndrangheta/Sacra Corona Unita)? R

E11) Quanta Indifferenza ti evoca la criminalità organizzata di stampo mafioso (Cosa Nostra/Camorra/’Ndrangheta/Sacra Corona Unita)?

B3) Denuncerei alle autorità competenti l’attività illecita di una persona appartenente alla criminalità organizzata di stampo mafioso (Cosa Nostra/Camorra/’Ndrangheta/Sacra Corona Unita), se ne fossi a conoscenza R

B4) Non mi ribellerei se una persona appartenente alla criminalità organizzata di stampo mafioso (Cosa Nostra/Camorra/’Ndrangheta/Sacra Corona Unita) mi chiedesse il pizzo

B7) Se vedessi una persona minacciata da qualcuno appartenente alla criminalità organizzata di stampo mafioso (Cosa Nostra/Camorra/’Ndrangheta/Sacra Corona Unita), denuncerei il fatto alle autorità competenti

B12) Parteciperei a manifestazioni contro la criminalità organizzata di stampo mafioso (Cosa Nostra/Camorra/’Ndrangheta/Sacra Corona Unita) R

B19) Sarei disposto a investire del denaro per organizzare iniziative contro la criminalità organizzata di stampo mafioso (Cosa Nostra/Camorra/’Ndrangheta/Sacra Corona Unita) R

B20) Non denuncerei un mio parente se venissi a conoscenza che ha collaborato con la criminalità organizzata di stampo mafioso (Cosa Nostra/Camorra/’Ndrangheta/Sacra Corona Unita)

B21) Denuncerei l’attività illecita della criminalità organizzata di stampo mafioso (Cosa Nostra/Camorra/’Ndrangheta/Sacra Corona Unita) anche se questo vuol dire andare contro alle persone del mio vicinato e ai miei amici R

C1) Le persone appartenenti alla criminalità organizzata di stampo mafioso (Cosa Nostra/Camorra/’Ndrangheta/Sacra Corona Unita) sono meno oneste delle altre persone R

C6) Alcuni valori trasmessi dalle famiglie appartenenti alla criminalità organizzata di stampo mafioso (Cosa Nostra/Camorra/’Ndrangheta/Sacra Corona Unita) sono condivisibili

C13) Le persone appartenenti alla criminalità di stampo mafioso (Cosa Nostra/Camorra/’Ndrangheta/Sacra Corona Unita) sono brave nelle loro attività

C15) La criminalità organizzata di stampo mafioso (Cosa Nostra/Camorra/’Ndrangheta/Sacra Corona Unita) impoverisce i territori in cui è presente R

C29) Un membro della criminalità organizzata di stampo mafioso (Cosa Nostra/Camorra/’Ndrangheta/Sacra Corona Unita) è una persona più furba delle altre

C30) Le persone appartenenti alla criminalità organizzata di stampo mafioso (Cosa Nostra/Camorra/’Ndrangheta/Sacra Corona Unita) pensano solo al proprio interesse R

C34) Le persone appartenenti alla criminalità organizzata di stampo mafioso (Cosa Nostra/Camorra/’Ndrangheta/Sacra Corona Unita) sono persone che sanno farsi rispettare

C35) La criminalità organizzata di stampo mafioso (Cosa Nostra/Camorra/’Ndrangheta/Sacra Corona Unita) offre opportunità lavorative che lo Stato non è in grado di dare

*R = reversed score

***Analyses on AIMS factor scores as predictors***

*Criterion-Predictive Validity*
The model with latent factor scores of the AIMS as predictors (χ^2^(1) = 23.98, *p* < .001) yielded similar results (b = 0.58, SE = 0.12, z-value = 4.67, *p* <.001), suggesting that the scores extracted from the latent factors are equally capable of predicting donation behavior.

*Comparing AIMS scores between Deep-rooted Mafia regions and rest of Italy*

*Frequentist approach*

The analysis on AIMS fscores confirmed results of the AIMS mean scores with even stronger results t(391) = -3.11, *p* = .002, Cohen’s d = -.31, 95% CI [-.51, -.11]), again with DRMR participants expressing more negative attitudes towards Mafias (M_DRMR_ = -0.13) compared to participants from the rest of Italy, M_Rest_ = 0.14).

*Bayesian approach*

The analysis on the AIMS fscores strongly supported the results obtained with the frequentist approach, with a BF_10_ = 11.46 indicating strong evidence in favor of a difference between DRMR participants (M_DRMR_ = -.13, 95% CI [-.26, -.00] and those form the rest of Italy (M_Rest_ = .14, 95% CI [.02, .25]. The error percentage was equal to 0.002%, which indicated a great stability in the algorithm used in the analysis. Here, across a wide range of widths the Bayes factor appeared to be less stable, ranging from 6.55 to 16.41, but still well above the accepted value for moderate evidence.

***Analyses with scores based on 7 response categories***

We performed the same frequentist (parametric and non-parametric) and Bayesian analyses on AIMS mean scores and AIMS fscores based on 7 instead of 6 response categories, yielding a similar pattern of results.
-Parametric independent sample t-test on AIMS mean scores: t(391) = -1.99, *p* = .048, Cohen’s d = -.20, 95% CI [-.40, -.00], MDRMR = 2.46, MRest = -.2.61.
-Parametric independent sample t-test on AIMS fscores: t(391) = -2.90, *p* = .004, Cohen’s d = -.29, 95% CI [-.49, -.09], M_DRMR_ = -.12, M_Rest_ = .13.
-Non-parametric Mann-Whitney U-test on AIMS mean scores: U = 16693.50, *p* = .02, r_rb_ = -.13, M_DRMR_ = 2.46, M_Rest_ = -.2.61.
-Non-parametric Mann-Whitney U-test on AIMS fscores: U = 15633.50, *p* = .001, r_rb_ = -.19, M_DRMR_ = -.12, M_Rest_ = .13.
-Bayesian independent sample t-test on AIMS mean scores: BF_10_ = 0.74, %error = 0.02, BF10 ranging from 0.40 to 1.65, M_DRMR_ = 2.46, 95% CI [2.34, 2.57], M_Rest_ = -.2.61, 95% CI [2.52, 2.72].
-Bayesian independent sample t-test on AIMS fscores: BF_10_ = 6.24, %error = 0.004, BF_10_ ranging from 3.52 to 9.48, MDRMR = -.12, 95% CI [-.25, -.00], MRest = .13, 95% CI [.02, .25].
-Bayesian non-parametric Mann-Whitney U-tests on AIMS scores: BF_10_ =0.83, W = 16693.50, rhat = 1.000, M_DRMR_ = 2.46, 95% CI [2.34, 2.57]), M_Rest_ = -.2.61, 95% CI [2.52, 2.72].
-Bayesian non-parametric Mann-Whitney U-tests on AIMS fscores: BF_10_ =4.23, W = 15633.50, rhat = 1.003, M_DRMR_ = -.12, 95% CI [-.25, -.00], M_Rest_ = .13, 95% CI [.02, .25].

***Frequentist non-parametric Mann-Whitney U-tests with scores based on 6 response categories***

Since the response scale was ordinal in nature, we performed two non-parametric Mann-Whitney U-tests). Confirming parametric analysis, the non-parametric tests on AIMS mean scores revealed a significant difference (U = 16554.50, *p* = .01), with DRMR participants having a more negative attitude towards Mafia compared to those from the Rest of Italy (M_DRMR_ = 2.43, M_Rest_ = 2.60; r_rb_ = -.14, 95% CI [-.25, -.03]).
Importantly, the analysis on AIMS fscores supported those findings yielding even stronger results (U = 15407.50, *p* < .001), with again DRMR participants having a more negative attitude towards Mafia compared to those from the Rest of Italy (M_DRMR_ = -0.13, M_Rest_ = 0.14; r_rb_ = -.20, 95% CI [-.31, -.09]).

***Bayesian non-parametric Mann-Whitney U-tests with scores based on 6 response categories***

To test the robustness of the frequentist non-parametric analysis, we performed two Bayesian non-parametric Mann-Whitney U-tests on AIMS scores and AIMS fscores.
Confirming Bayesian parametric analysis, we did not find enough evidence for a difference on AIMS scores (BF_10_ =1.12, W = 16554.50, rhat = 1.005) between DRMR participants (M_DRMR_ = 2.43, 95% CI [2.32, 2.54] and those from the rest of Italy (M_Rest_ = 2.60, 95% CI [2.49, 2.70] but strong evidence for an effect on AIMS fscores (BF_10_ =8.04, W = 15407.50, rhat = 1.002), with DRMR participants (M_DRMR_ = -.13, 95% CI [-.26, -.00]) having more negative attitudes compared to participants from the rest of Italy (M_Rest_ = .14, 95% CI [.02, .25]).
